# A new species of *Socarnes* Boeck, 1871 (Crustacea, Amphipoda, Lysianassidae) from Korean waters

**DOI:** 10.3897/zookeys.357.6372

**Published:** 2013-11-29

**Authors:** Young-Hyo Kim, Ed A. Hendrycks

**Affiliations:** 1 Canadian Museum of Nature, Research and Collections, P.O. Box 3443, Station D, Ottawa, Canada K1P 6P4

**Keywords:** Amphipoda, lysianassid, *Socarnes*, new species, Korea, key, taxonomy

## Abstract

A new species of lysianassid amphipod belonging to the genus *Socarnes* Boeck was collected from Korean coastal waters. This is the first record of the genus *Socarnes* from Korea. The new species is fully illustrated and extensively compared with related species. A key to *Socarnes* speciesis provided.

## Introduction

The subfamily Lysianassinae Dana, 1849 is a large group of the family Lysianassidae, with 29 genera. Among these genera, *Socarnes* has close affinity to the *Concarnes*, *Socarnoides*, *Socarnella*, *Socarnopsis*, and *Socarnella* by having a simple gnathopod 1 and cleft telson. However, the *Socarnes* is further characterized by the protruded upper lip, apically serrated palp of maxilla 1, and biarticulated outer ramus of uropod 3.

Species of *Socarnes* are widely distributed and are found in shallow to deep water, from the Arctic to the tropical South Pacific including temperate regions (Mediterranean and East Asia). Ecologically, they are well known scavengers (Lowry and Stoddart 1994; Hall-Spencer and Bamber 2007). The genus is relatively small, being comprised of 10 species. Hitherto, only two species of the subfamily Tryphosinae belonging to the family Lysianassidae, *Orchomenella obtusa* (Sars, 1895) and *Orchomenella japonica* Gurjanova, 1962 have been recorded in Korea (KSSZ 1997, Jung and Kim 2008), even though the family Lysianassidae is one of the most speciose. This is the first record of the genus *Socarnes* from Korea and brings the total recorded number of lysianassid species to three from Korea. Many more are to be expected with increased sampling in this area, considering the high diversity of the group. A key to the world *Socarnes* species is also provided.

## Material and methods

Specimens were collected with a hand-net from various algae, seagrass and coral rubble from shallow waters of Gyeongpo, Tongyeong-si, Korea. The specimens were fixed with 80% ethyl alcohol and dissected in glycerol on Cobb’s aluminum hollow slides. Permanent mounts were made using polyvinyl lactophenol with lignin pink added. Drawings and measurements were performed with the aid of a drawing tube, mounted on an Olympus SZX 12 stereomicroscope and Olympus BX 51 interference contrast compound microscope. The body length was measured from the tip of rostrum to the end of the telson, along the dorsal parabolic line of the body. Nomenclature of the terms ‘tooth’, ‘spine’ and ‘seta’ follows Barnard and Karaman (1991) and terminology of the setae of the mandibular palp follows Lowry and Stoddart (1993). Type specimens are deposited at the National Institute of Biological Resources (NIBR), Incheon, Korea, Department of Biological Science, Dankook University (DKU), Cheonan, Korea and the Canadian Museum of Nature (CMN), Ottawa, Canada.

## Taxonomy

### Genus Socarnes

Boeck, 1871

http://species-id.net/wiki/Socarnes

(Korean Name: Min-son-gin-pal-yeop-sae-u-sok, new)

#### Type species.

*Lysianassa vahlii* Krøyer, 1838

#### Diagnosis.

Lateral cephalic lobe acutely projecting. Mouthparts forming quadrate bundle. Upper lip protruded strongly beyond epistome with rounded lobe. Maxilla 1, palp serrate apically. Gnathopod 1 simple. Uropod 2, inner ramus with or without notch. Uropod 3, outer ramus biarticulate, longer than inner ramus. Telson cleft.

#### Species composition.

The genus contains 11 species as follows: *Socarnes allectus* Andres, 1981; *Socarnes bidenticulatus* (Bate, 1858); *Socarnes erythrophthalmus* Robertson, 1892; *Socarnes filicornis* (Heller, 1866); *Socarnes hartmani* Hurley, 1963; *Socarnes rurutu* Lowry & Stoddart, 1994; *Socarnes septimus* Griffiths, 1975; *Socarnes tiendi* Lowry & Stoddart, 1994; *Socarnes tongyeongensis* sp. n. (this study); *Socarnes tuscarora* Lowry & Stoddart, 1994 and *Socarnes vahlii* (Krøyer, 1838).

### Socarnes tongyeongensis sp. n.

http://zoobank.org/2E99DBF6-302F-49BC-9605-7562A2DAF5FC

http://species-id.net/wiki/Socarnes_tongyeongensis

**Figure 1.**
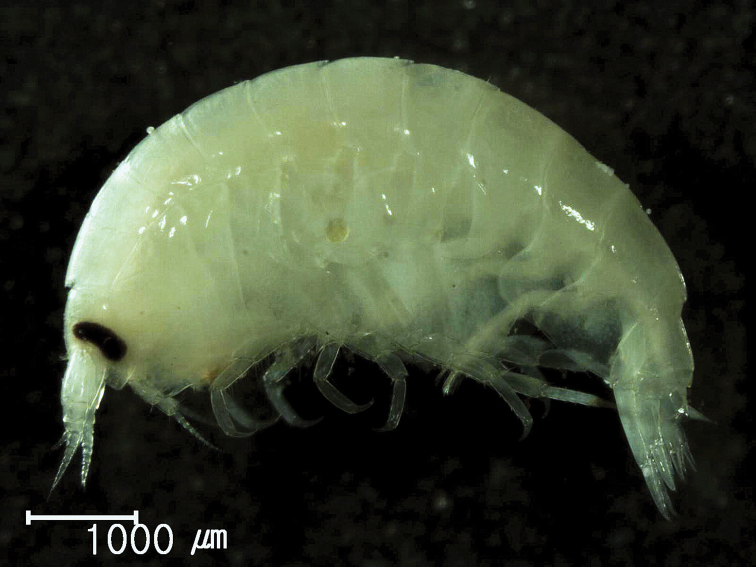
*Socarnes tongyeongensis* sp. n., female, 8.8 mm, Gyeongpo, Pungwha-ri, Sanyang-eup, Tongyeong-si, Korea.

**Figure 2.**
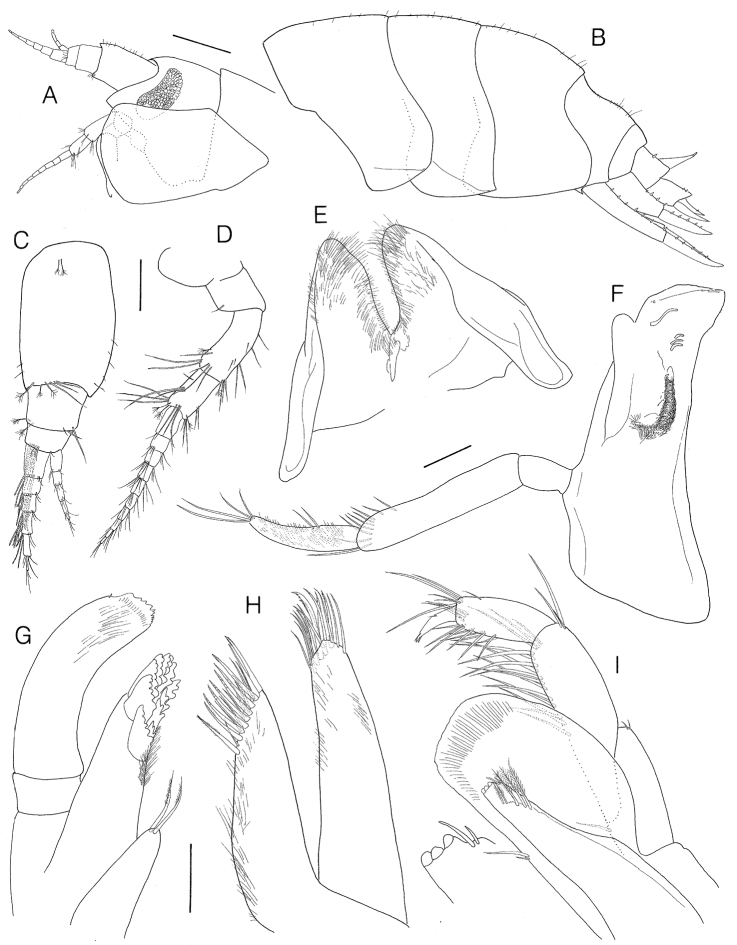
*Socarnes tongyeongensis* sp. n., holotype, female, 9.3 mm, Gyeongpo, Pungwha-ri, Sanyang-eup, Tongyeong-si, Korea. **A** head **B** Pleonites **C** antenna 1 **D** antenna 2 **E** lower lip **F** left mandible **G** maxilla 1 **H** maxilla 2 **I** maxilliped. Scale bars: 0.5 mm (**A**, **B**), 0.2 mm (**C**, **D**), 0.1 mm (**E–I**).

**Figure 3.**
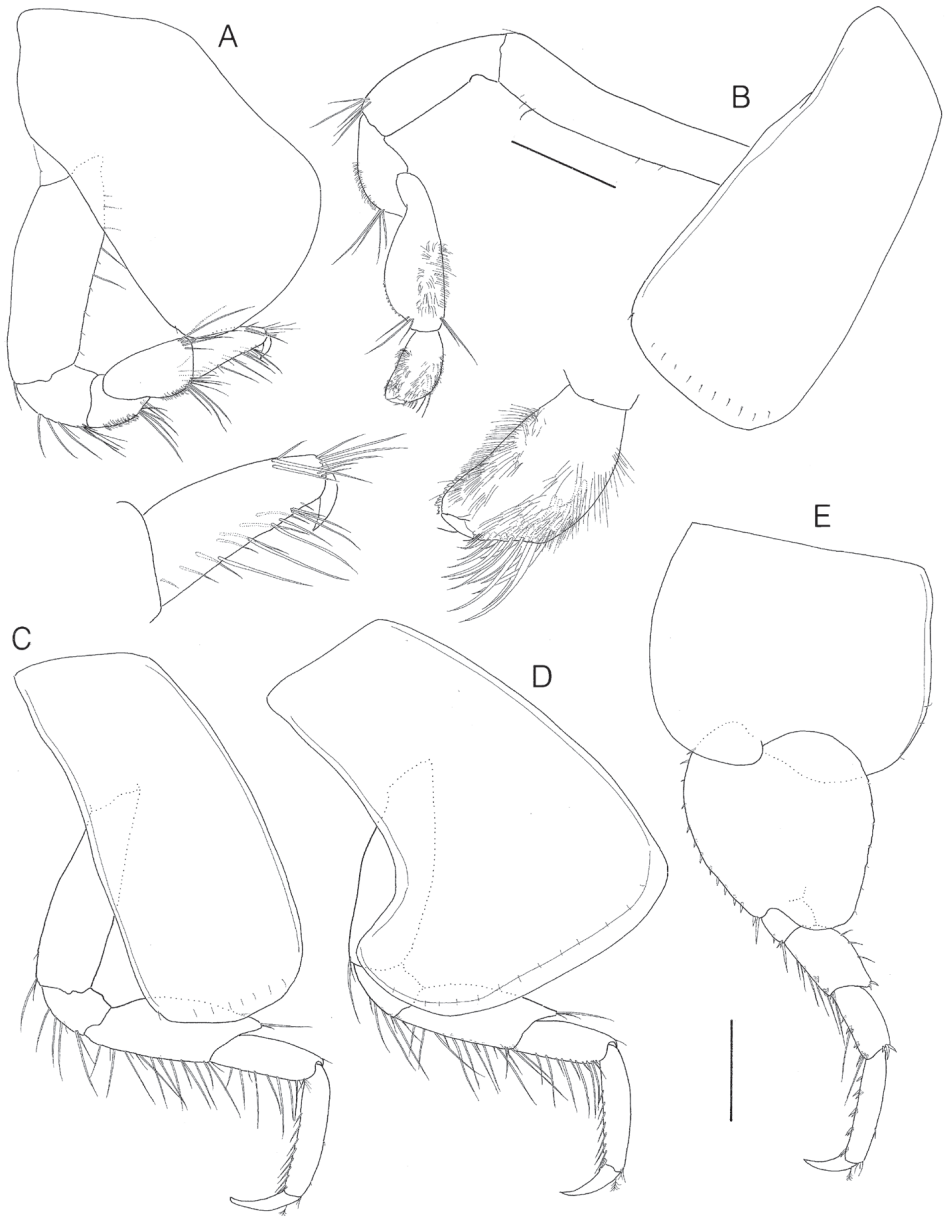
*Socarnes tongyeongensis* sp. n., holotype, female, 9.3 mm, Gyeongpo, Pungwha-ri, Sanyang-eup, Tongyeong-si, Korea. **A** gnathopod 1 **B** gnathopod 2 **C** pereopod 3 **D** pereopod 4 **E** pereopod 5. Scale bars: 0.4 mm.

**Figure 4.**
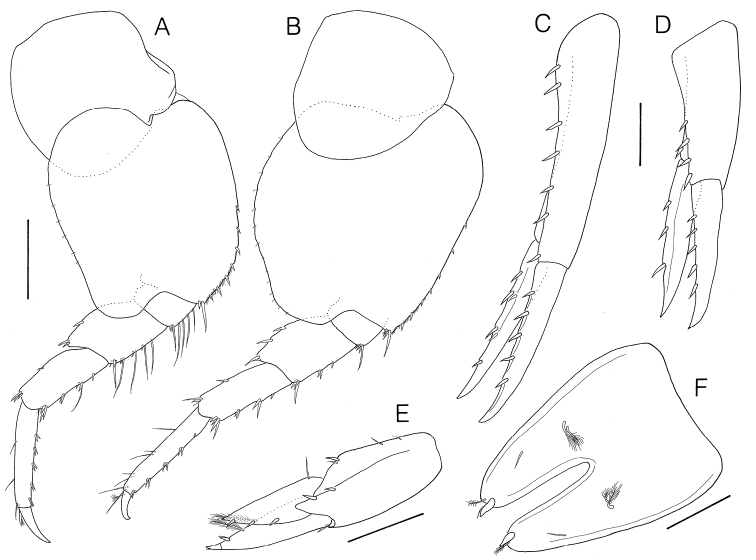
*Socarnes tongyeongensis* sp. n., holotype, female, 9.3 mm, Gyeongpo, Pungwha-ri, Sanyang-eup, Tongyeong-si, Korea. **A** pereopod 6 **B** pereopod 7 **C** uropod 1 **D** uropod 2 **E** uropod 3 **F** Telson. Scale bars: 0.4 mm (**A**, **B**), 0.2 mm (**C–E**), 0.1 mm (**F**).

Korean name: Tong-yeong-gin-pal-yeop-sae-u, new Figures 1 –4

#### Type material.

Holotype: female, 9.3 mm, NIBRIV0000282400, Gyeongpo, Pungwha-ri, Sanyang-eup, Tongyeong-si, Gyeongsangnam-do, Korea, 34°49'47"N, 128°22'21"E, Y.H. Kim, 24 August 2005. Paratypes: two females, 7.2, 11.3 mm, CMNC 2013-0003, same station data as holotype; six females, 6.3–10.7 mm, DKU 201301, same station data as holotype.

#### Description.

**Holotype**, **female**, NIBRIV0000282400.

Body (Fig. 2A) 9.3 mm long. Eye medium, reniform, black. Lateral cephalic lobe apically triangular, pointed. Upper lip protruded beyond epistome in large rounded lobe. Pereonites 1–7 dorsally smooth. Pleonites 1–3 (Fig. 2B) dorsally smooth, with very fine setae. Epimeron 2 with small posteroventral tooth. Epimeron 3 with rounded-quadrate posteroventral corner. Epimera 2–3 slightly deeper than epimeron 1. Urosomite 1 shallowly concave anterodorsally, with fine setae. Urosomite 3 with 1 small dorsal spine on each side.

Antenna 1 (Fig. 2C) short, 1.46 × head, weakly setose; peduncular article 1 stout, 0.6 × as wide as long; peduncular article 3 short, 0.67 × flagellum article 1, with transverse row of aesthetascs distomedially; length ratio of peduncular articles 1–3 = 1.00: 0.32: 0.16; flagellum 6-articulate, slightly shorter than peduncle, each article bearing long aesthetascs; accessory flagellum 5-articulate, 0.54 × primary flagellum.

Antenna 2 (Fig. 2D) slender, moderately setose, subequal in length to antenna 1; peduncular article 4 2.44 × article 5; flagellum 10-articulate, proximal article slightly shorter than peduncular article 5, subequal in length to second and third articles combined.

Lower lip (Fig. 2E) with broad outer lobe covered with patch of pubescence medially, mandibular process thin and blunt apically.

Left mandible (Fig. 2F) incisor simple, smooth, blunt apically; lacinia mobilis simple, slender, and curved; accessory setal row with 3 small blunt spines; molar elongate and forms a setose ridge; palp triarticulate, set below molar level, proximal segment short, article 2 elongate 1.63 × article 3, with 8 A2 setae and 1 E2 seta, distal article covered with patch of pubescence laterally, with 2 D3 setae and 4 unequal E3 setae.

Maxilla 1 (Fig. 2G) inner plate small, narrowing distally with 2 weakly plumose apical setae; outer plate armed with 11 (3–6 multi dentate) spine-teeth in 7/4 arrangement; palp curved, broad, biarticulate, proximal segment short, subrectangular, distal one extending beyond end of outer lobe, serrate apically without terminal setae.

Maxilla 2 (Fig. 2H) inner plate with dense pubescence medially, apical margin with 2 rows of simple or pectinate setae; outer plate subequal in width to inner and slightly longer, with simple and pectinate setae apically.

Maxilliped (Fig. 2I) inner plate slender, reaching beyond article 1 of palp, with 3 apical nodular spines and an oblique row of 5 plumose setae; outer plate broad, subquadrate, distal margin subtruncate, reaching less than distal end of article 2 of palp, lacking apical setae or spines except 2 unequal simple setae medially; palp slender, 4-articulate, article 2 elongate, medial margin with 15 simple setae, article 3 subrectangular; distal article falcate, 0.55 × article 3.

Gnathopod 1 (Fig. 3A) coxa large, subquadrate, 0.56 × as wide as long, anterior margin slightly concave, broadly rounded anteroventrally; basis subrectangular, width 0.37 × length, subequal in length to merus - dactylus combined, with 5 simple setae anteriorly; merus and carpus with cluster of simple setae posterodistally and denticulate patches posteriorly; propodus characteristic in form, simple, gradually narrowing distally, 0.85 × carpus, with long simple setae on the medial and posterior margins; dactylus falcate, short.

Gnathopod 2 (Fig. 3B) coxa subrectangular, width 0.45 × length, gradually broadened distally; basis linear, elongate; ischium elongate, subequal in length to carpus, with cluster of 5 simple setae posterodistally; carpus broadly convex posteriorly, anterolateral surface with patch of spinules; propodus subovate, minutely chelate, rather elongated, narrowing anterodistally, posterior margin straight, length 0.51 × carpus, with surface covered by tiny spinules, medially with 4 rows of simple setae, palm obtuse; dactylus small, short, stubby, slightly less than palmar corner.

Pereopod 3 (Fig. 3C) coxa similar in shape but slightly narrower (× 0.9) than that of gnathopod 2; ischium to carpus with simple setae on posterior margins; propodus subrectangular, with a row of 8 stiff setae posteriorly and 2 small spines posterodistally; dactylus length 0.5 × propodus.

Pereopod 4 (Fig. 3D) similar to pereopod 3 except coxa broader (× 1.78) than that of pereopod 3, posterodistal lobe strong, posterior margin strongly excavate.

Pereopod 5 (Fig. 3E) coxa large, width 1.21 × length, equilobate, posterior lobe broader than anterior; basis subovate, roundly expanded posteriorly, width 0.84 × length, posterodistal lobe reaching beyond ischium, with row of 12 spines anteriorly and unequal triad spines anterodistally; merus subequal in length to carpus, with long setae and small spines anteriorly; propodus subrectangular, 1.56 × carpus, with 5 clusters of spines anteriorly; dactylus falcate, 0.44 × propodus.

Pereopod 6 (Fig. 4A) coxa quadrangular, with rounded corners, subequal in length and width, weakly bilobate; basis subovate, broadly rounded, expanded posteriorly, width 0.84 × length, anterodistal margin with row of spines, posterior margin straight; ischium lined with long setae anteriorly; merus broad, width 0.65 × length, subequal in length to carpus, lined with 4 long setae anteriorly and 1 long spine anterodistally; propodus subrectangular, 1.17 × carpus, with 5 clusters of 2 spines on anterior margin.

Pereopod 7 (Fig. 4B) similar to pereopod 6, but coxa nonlobate, basis larger than that of pereopod 6, more broadly rounded, weakly serrulate posteriorly and with slight posterodistal truncation; anterior margins of ischium and merus lacking long setae.

Uropod 1 (Fig. 4C) peduncle subrectangular, 1.43 × outer ramus, with row of 6 dorsolateral spines, 1 apicolateral spine; outer ramus subequal in length to inner, dorsolateral margin with 5 spines, inner ramus with 4 dorsolateral and 2 medial spines.

Uropod 2 (Fig. 4D) peduncle subequal in length to outer ramus, with row of 4 dorsolateral and 1 apicomedial spines; rami subequal in length, outer with row of 5 dorsolateral spines, inner ramus with weak constriction and 3 dorsolateral spines.

Uropod 3 (Fig. 4E) peduncle longer than outer ramus, with subacute distolateral flange, 2 dorsolateral and 1 dorsomedial spines; rami lanceolate; outer ramus biarticulate, 1.14 × inner ramus, proximal article with 2 plumose setae and 1 spine laterally, distal article short, 0.12 × proximal article; inner ramus with 1 proximomedial seta and 3 lateral spines.

Telson (Fig. 4F) subtriangular, width 1.33 × length, moderately cleft (50%), with two pairs of penicillate setae and 2 setules on dorsal surface, each lobe rounded with 1 spine and 1 penicillate seta apically.

Male. Unknown.

#### Remarks.

The new species is morphologically similar to *Socarnes vahlii* (Krøyer, 1838) in size, in the short dactylus of gnathopod 2 and constricted inner ramus of uropod 2. However, the new species is obviously distinguished from *Socarnes vahlii* (different characters of *Socarnes vahlii* in brackets) by the combination of the following features: 1) gnathopod 2, propodus subequal to half the length of carpus and narrowing anterodistally (vs less than half the length of carpus and broadened distally); 2) epimeron 3 with rounded posterior corner (vs obtusely truncated posterior corner); 3) telson broad, length 1.3 × width, moderately cleft, about half of its length (vs narrow, length 1.6 × width, cleft > the middle of its length). The redescription of *Socarnes vahlii* (Krøyer, 1838) by Nagata (1965), which is the only species of the genus recorded in Japan, was brief and lacked illustrations. He mentioned that his specimens were in agreement with Sars’ figures (1895, Pl. 16, fig. 2) except for the shape of the posteroventral corner of epimeron 3, which is similar to *Socarnes erythrophthalmus* Robertson, 1892 and *Socarnes tongyeongensis* sp. n. Our specimens show morphological similarities to the redescription of Nagata (1965). However, as we have not had the opportunity to examine the material of Nagata (1965), we cannot confidently determine whether Nagata’s specimens are conspecific with ours, despite the geographic proximity. Therefore at this time we conservatively maintain Nagata’s specimens as *Socarnes vahlii* until a detailed, comparative study can be made on the type material. Given the great geographic distance however, we would expect Nagata’s specimens to differ from *Socarnes vahlii*, as that species was recorded from Greenland, North Western Atlantic and Arctic Oceans.

#### Etymology.

The species name is derived from the type locality, Tongyeong-si located on the south coast of Korea.

#### Distribution.

Gyeongpo, Pungwha-ri, Sanyang-eup, Tongyeong-si, Gyeongsangnam-do, Korea.

### Key to species of *Socarnes*

**Table:** 

1	Body small, < 5 mm	2
–	Body medium to large, > 5 mm	3
2	Telson cleft 50% of its length	*Socarnes erythrophthalmus* Robertson, 1892
–	Telson cleft > 50% of its length	*Socarnes septimus* Griffiths, 1975
3	Epimeron 3 with posteroventral tooth	*Socarnes hartmani* Hurley, 1963
–	Epimeron 3 without posteroventral tooth	4
4	Body large, > 30 mm; epimeron 3 with midposterior process	*Socarnes bidenticulatus* (Bate, 1858)
–	Body medium, < 30 mm; epimeron 3 without midposterior process	5
5	Telson deeply cleft, > 65% of its length; maxilliped, outer plate reaching beyond palp article 2	6
–	Telson moderately cleft, < 65% of its length; maxilliped, outer plate < palp article 2	7
6	Lateral cephalic lobe subacute	*Socarnes allectus* Andres, 1981
–	Lateral cephalic lobe rounded	*Socarnes filicornis* (Heller, 1866)
7	Gnathopod 2, dactylus reaching palmar corner; epimeron 3 posteroventrally narrowly rounded	8
–	Gnathopod 2, dactylus short, < palmar corner; epimeron 3 posteroventrally rounded-quadrate	10
8	Gnathopod 2, palm strongly concave	*Socarnes tiendi* Lowry & Stoddart, 1994
–	Gnathopod 2, palm obtuse	9
9	Pereopod 4, posterior margins of merus-carpus weakly setose; pereopod 7, basis with rounded posteroventral margin; telson cleft about 50% of its length	*Socarnes rurutu* Lowry & Stoddart, 1994
–	Pereopod 4, posterior margins of merus-carpus strongly setose; pereopod 7, basis with truncate posteroventral margin; telson cleft > 60% of its length	*Socarnes tuscarora* Lowry & Stoddart, 1994
10	Gnathopod 2, propodus medially broad, narrowing anterodistally; telson broad, length 1.3 × width, cleft about 50% of its length	*Socarnes tongyeongensis* sp. n.
–	Gnathopod 2, propodus stubby, broadened distally; telson narrow, length 1.6 × width, cleft >50% of its length	*Socarnes vahlii* (Krøyer, 1838)

## Supplementary Material

XML Treatment for Socarnes

XML Treatment for Socarnes tongyeongensis
